# Differential Responses to Sigma-1 or Sigma-2 Receptor Ablation in Adiposity, Fat Oxidation, and Sexual Dimorphism

**DOI:** 10.3390/ijms231810846

**Published:** 2022-09-16

**Authors:** Jing Li, Elisa Félix-Soriano, Katherine R. Wright, Hongtao Shen, Lisa A. Baer, Kristin I. Stanford, Lian-Wang Guo

**Affiliations:** 1 Department of Surgery, School of Medicine, University of Virginia, Charlottesville, VA 22908, USA; 2 Department of Physiology & Cell Biology, College of Medicine, Wexner Medical Center, Davis Heart and Lung Research Institute, The Ohio State University, Columbus, OH 43210, USA; 3 Department of Biochemistry and Molecular Genetics, University of Virginia, Charlottesville, VA 22908, USA; 4 Robert M. Berne Cardiovascular Research Center, University of Virginia, Charlottesville, VA 22908, USA

**Keywords:** sigma receptors, obesity, fat mass and lean mass, fatty acid oxidation, insulin tolerance, sexual dimorphism

## Abstract

Obesity is increasing at epidemic rates across the US and worldwide, as are its co-morbidities, including type-2 diabetes and cardiovascular disease. Thus, targeted interventions to reduce the prevalence of obesity are of the utmost importance. The sigma-1 receptor (S1R) and sigma-2 receptor (S2R; encoded by *Tmem97*) belong to the same class of drug-binding sites, yet they are genetically distinct. There are multiple ongoing clinical trials focused on sigma receptors, targeting diseases ranging from Alzheimer’s disease through chronic pain to COVID-19. However, little is known regarding their gene-specific role in obesity. In this study, we measured body composition, used a comprehensive laboratory-animal monitoring system, and determined the glucose and insulin tolerance in mice fed a high-fat diet. Compared to *Sigmar1+/+* mice of the same sex, the male and female *Sigmar1−/−* mice had lower fat mass (17% and 12% lower, respectively), and elevated lean mass (16% and 10% higher, respectively), but S1R ablation had no effect on their metabolism. The male *Tmem97−/−* mice exhibited 7% lower fat mass, 8% higher lean mass, increased volumes of O_2_ and CO_2_, a decreased respiratory exchange ratio indicating elevated fatty-acid oxidation, and improved insulin tolerance, compared to the male *Tmem97+/+* mice. There were no changes in any of these parameters in the female *Tmem97−/−* mice. Together, these data indicate that the S1R ablation in male and female mice or the S2R ablation in male mice protects against diet-induced adiposity, and that S2R ablation, but not S1R deletion, improves insulin tolerance and enhances fatty-acid oxidation in male mice. Further mechanistic investigations may lead to translational strategies to target differential S1R/S2R regulations and sexual dimorphism for precision treatments of obesity.

## 1. Introduction

The obesity epidemic continues to increase at alarming rates in the US and around the world. Obesity is a complex disease that can lead to other major complications, most notably type 2 diabetes and cardiovascular disease [1]. Despite extensive public health efforts to prevent and combat obesity, no clear treatment is available. This underscores the necessity of discovering novel druggable targets as a basis for developing effective new treatments.

The sigma-1 receptor (S1R) and sigma-2 receptor (S2R) constitute a unique class of drug-binding sites [2] which share ligands, yet differ in pharmacology [3,4]. They were initially characterized as opioid receptors, but later clarified to be non-opioid receptors encoded by two different genes [5,6,7]. Most recently, they were found to assume distinct structures [8,9]. With endogenous ligands still elusive [10], both S1R and S2R remain orphan receptors. S1R has been studied in a range of diseases, including cancer, heart failure, psychological disorders, and neurodegeneration [3]. Currently, S1R ligands are undergoing human trials to treat neuropathic pain, Alzheimer’s disease, ischemic stroke, and COVID-19 [11,12,13,14]. While evidence indicates S1R’s involvement in lipid metabolism [15,16,17,18], it remains unclear whether S1R confers a novel interventional target in obesity [15].

The gene encoding S2R was recently identified as TMEM97, a scarcely studied gene [5], thus the TMEM97 gene-specific knowledge on S2R is still very limited. S2R has been pharmacologically targeted for decades, mainly in preclinical cancer treatment/imaging and research into psychiatric disorders. Clinical trials are ongoing to test the efficacy of S2R ligands for treating schizophrenia and Alzheimer’s disease [19]. Interestingly, S2R, or TMEM97, is reportedly involved in cholesterol homeostasis [20,21,22]. However, the role of this receptor in obesity is under-explored.

We previously found that the ablation of S1R (*Sigmar1* KO) negated the body-weight gain of mice on a high-fat diet (HFD) and impaired adipogenesis in vitro [15]. Given the aforementioned link between S1R and S2R and lipid biology, we explored the role of S1R and S2R in obesity by applying the unique power of the comprehensive laboratory-animal monitoring system (CLAMS) to live animals on HFD. The main objective of this study was to determine the impact of S1R or S2R ablation on diet-induced adiposity, metabolism, and energy expenditure in male and female mice.

## 2. Results

### 2.1. S1R Ablation Protects against Adiposity in Male and Female Mice Fed on HFD

**Figure 1:**
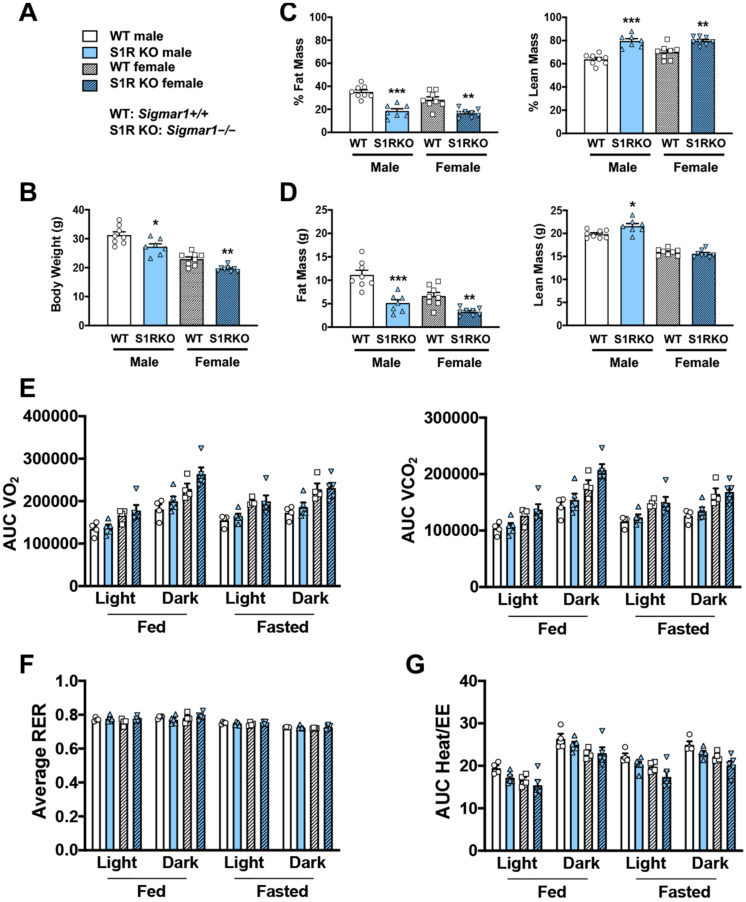
S1R ablation reduces body-fat mass and increases lean mass in HFD-fed male and female mice. All measurements were performed after 6 weeks of HFD. (**A**). Legends for (**B**–**G**). S1R WT: *Sigmar1+/+*. S1R KO: *Sigmar1−/−*. (**B**). Body weight. (**C**). Body composition (% mass vs. total mass). (**D**). Body composition (mass in grams). (**E**). Metabolism. Data were normalized to body weight. (**F**). Respiratory exchange ratio (RER). (**G**). Heat/EE (energy expenditure). Data were normalized to body weight. Each dot (data point, please see the circle, triangle, and square symbols) on the plots represents the area under the curve (AUC) or average (for RER) of each 12-h time period for each individual animal. Statistical analysis: One-way (**B**–**D**) or two-way (**E**–**G**) ANOVA with Bonferroni’s multiple comparisons test (* *p* < 0.05, ** *p* < 0.01, *** *p* < 0.001), S1R KO vs. WT, *n* = 7 or 8 mice in (**B**–**D**) or *n* = 4 or 5 mice in (**E**–**G**). Data are presented as means ± SE.

To determine whether body weight or body composition were altered in S1R KO mice, 8–10-week-old male and female S1R wild-type (WT) and KO mice were placed on a HFD, and measurements were performed 6 weeks after feeding with high fat (Figure 1). S1R ablation resulted in reduced body weight in male and female mice (Figure 1A,B and Figure S1, Tables S1 and S2). The male *Sigmar1−/−* mice had 17% less fat mass and 16% higher lean mass; similarly, the female *Sigmar1−/−* mice had 12% less fat mass and 10% higher lean mass (Figure 1C,D). To determine the mechanism behind this difference, metabolic cages were used to measure the volume of O_2_ (VO_2_), the volume of CO_2_ (VCO_2_), the respiratory exchange ratio (RER), and energy expenditure. There was no difference between the *Sigmar1+/+* and *Sigmar1−/−* mice (Figure 1E–G). There was no difference in food intake between the groups (Figure S2). These results indicate that in the absence of S1R, male and female mice develop a phenotype with increased lean mass and reduced adiposity, even in the presence of an obesity-inducing HFD.

### 2.2. S2R Deletion Reduces Adiposity and Increases Fatty-Acid Oxidation in Male, but Not Female, Mice Fed on HFD

**Figure 2:**
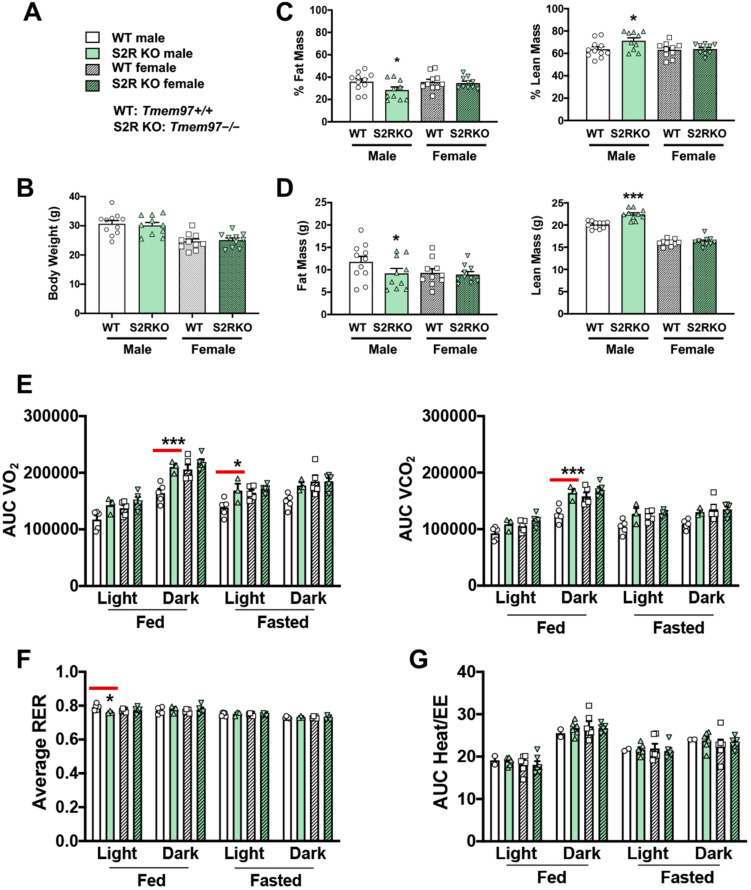
S2R ablation reduces fat mass and increases lean mass in male, but not female, mice fed on HFD. All measurements were performed after 6 weeks of HFD. (**A**). Legends for (**B**–**G**). S2R WT: *Tmem97+/+.* S2R KO: *Tmem97−/−.* (**B**). Body weight. (**C**). Body composition (% mass vs. total mass). (**D**). Body composition (mass in grams). (**E**). Metabolism. Data were normalized to body weight. (**F**). Respiratory exchange ratio (RER). (**G**). Heat/EE. Data were normalized to body weight. Each dot (data point, please see the circle, triangle, and square symbols) on the plots represents the area under the curve (AUC) or average (for RER) of each 12-h time period for each individual animal. Statistical analysis: One-way (**B**–**D**) or two-way (**E**–**G**) ANOVA with Bonferroni’s multiple comparisons test (* *p* < 0.05, *** *p* < 0.001), S2R KO vs. WT (the comparison indicated by a red line), *n* = 10 or 11 mice in (**B**–**D**) or *n* = 4 or 5 mice in (**E**–**G**). Data are presented as means ± SE.

To determine the influence of S2R/TMEM97 ablation on body composition, 8–10-week-old male and female *Tmem97+/+* and *Tmem97−/−* mice were placed on an HFD and their body composition was measured after 6 weeks of high-fat diet (Figure 2). There was no difference in body weight between the *Tmem97−/−* and *Tmem97+/+* groups (male or female, Figure 2A,B and Figure S3, Tables S3 and S4). However, in the male *Tmem97−/−* group, fat mass decreased by 7% and lean mass increased by 8% compared to male *Tmem97+/+* mice (Figure 2C,D) and independent of changes in food intake (Figure S2). Interestingly, these changes did not occur in the female *Tmem97−/−* mice, indicating a sexually dimorphic response.

We then utilized metabolic cages to determine whether the changes in body composition (*Tmem97−/−* vs. *Tmem97+/+*) were related to altered metabolism. The male *Tmem97−/−* mice showed increased consumption of O_2_ in dark-fed and light-fasted conditions, and increased CO_2_ production in the dark-fed condition compared to the male *Tmem97+/+* mice (Figure 2E). Consistent with these findings, the RER was reduced in the male *Tmem97−/−* (vs. *Tmem97+/+*) mice in the light-fed condition (Figure 2F), indicating increased lipid oxidation. These changes were independent of the changes in energy expenditure (Figure 2G). There were no differences between the female *Tmem97−/−* mice and the female *Tmem97+/+* mice. These data indicate that S2R/TMEM97 ablation in male mice results in reduced adiposity, increased lean body mass, and reduced RER, which can be at least partially explained by heightened fatty acid oxidation. Moreover, these changes were not observed in the female mice, demonstrating a sexually dimorphic phenotype.

### 2.3. S2R Ablation in Male Mice Improves Insulin Tolerance

**Figure 3:**
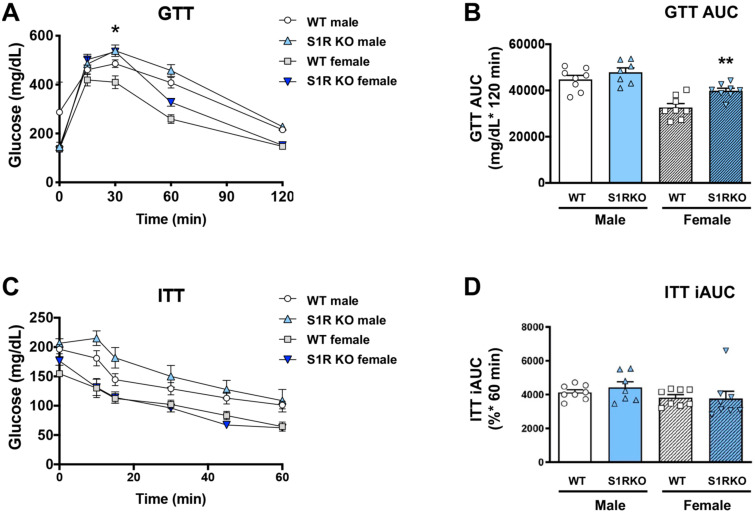
S1R ablation impairs glucose tolerance in female mice fed on HFD. GTT and ITT assays were performed after 6 weeks of HFD. (**A**). Time course of blood-glucose levels following i.p. injection of glucose (2.0 g/kg body weight). (**B**). Cumulative blood-glucose levels at 120 min after glucose injection. (**C**). Time course of blood glucose levels following i.p. injection of insulin (1 U/kg body weight). (**D**). Cumulative blood-glucose levels at 120 min after insulin injection. Statistical analysis: Two-way ANOVA with Bonferroni’s multiple-comparisons test (* *p* < 0.05, ** *p* < 0.01), S1R KO vs. WT, *n* = 7 or 8 mice. In (**B**,**D**), each dot (data point, please see the circle, triangle, and square symbols) represents the area under the curve (AUC) for each individual animal. Data are presented as means ± SE. iAUC: Inverse AUC, normalized to baseline levels.

Since obesity is closely associated with glucose intolerance, we next determined whether these sigma-receptor knockout mice had altered glucose or insulin tolerance after 6 weeks of HFD. No effect of glucose tolerance was observed in the male *Sigmar1−/−* mice, and glucose tolerance was impaired in *Sigmar1−/−* female mice (Figure 3A,B). The insulin tolerance did not differ between the groups (Figure 3C,D).

**Figure 4:**
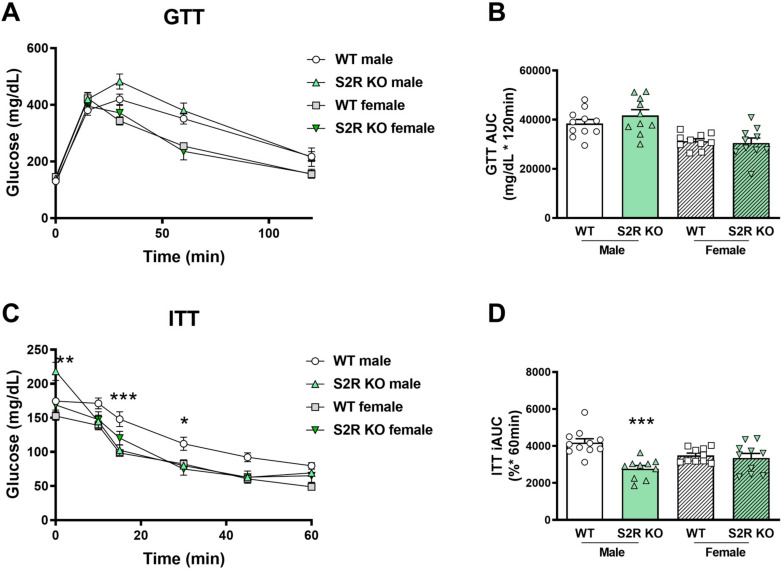
S2R ablation increases insulin tolerance in male, but not female, mice fed on HFD. GTT and ITT assays were performed after 6 weeks of HFD. (**A**). Time course of blood-glucose levels following i.p. injection of glucose (2.0 g/kg body weight). (**B**). Cumulative blood-glucose levels at 120 min after glucose injection. (**C**). Time course of blood-glucose levels following i.p. injection of insulin (1 U/kg body weight). (**D**). Cumulative blood-glucose levels at 120 min after insulin injection. Statistical analysis: Two-way ANOVA with Bonferroni’s multiple-comparisons test (* *p* < 0.05, ** *p* < 0.01, *** *p* < 0.001), S2R KO vs. WT, *n* = 10 or 11 mice. In (**B**,**D**), each dot (data point, please see the circle, triangle, and square symbols) represents the area under the curve (AUC) for each individual animal. Data are presented as means ± SE. iAUC: Inverse AUC, normalized to baseline levels.

There was no effect on glucose tolerance in the *Tmem97−/−* compared to the *Tmem97+/+* mice (male or female, Figure 4A,B), but insulin tolerance was improved in the male *Tmem97−/−* mice compared to the male *Tmem97+/+* mice (Figure 4C,D).

Taken together, these data indicate the role of S1R ablation in the impairment of glucose tolerance in female mice, and the role of S2R/TMEM97 ablation in improving insulin tolerance in male mice.

### 2.4. Sigma-Receptor Ablation Alters the Expression of Genes Involved in Lipolysis and Fatty-Acid Oxidation

**Figure 5:**
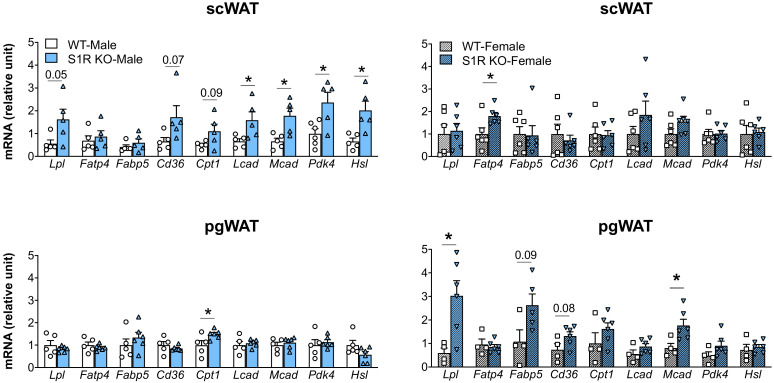
Effect of S1R ablation on the expression of genes involved in lipid metabolism and adipogenesis. Samples were collected from animals that were euthanized at the end point of 12 weeks after HFD feeding. Statistical analysis: Multiple *t*-test (* *p* < 0.05), S1R KO vs. WT, *n* = 5 or 6 mice. Each dot (data point, please see the circle, triangle, and square symbols) on the plots represents the relative gene-expression value for each individual animal. Data are presented as means ± SE.

**Figure 6:**
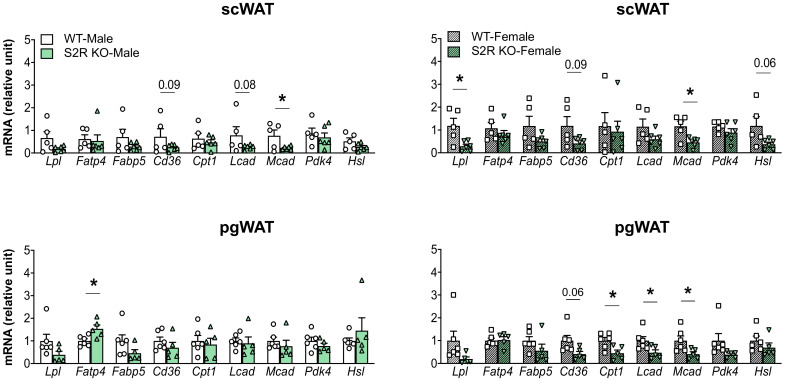
Effect of S2R ablation on the expression of genes involved in lipid metabolism and adipogenesis. Samples were collected from animals that were euthanized at the end point of 13 weeks after HFD feeding. Statistical analysis: Multiple *t*-test (* *p* < 0.05), S2R KO vs. WT, *n* = 5 or 6 mice. Each dot (data point, please see the circle, triangle, and square symbols) on the plots represents the relative gene-expression value for each individual animal. Data are presented as means ± SE.

The expression of the genes involved in lipolysis and fatty-acid oxidation was measured in the *Sigmar1−/−* (vs. *Sigmar1+/+*) mice. In the male mice, *Lcad, Mcad*, *Pdk4*, and *Hsl* were increased in the subcutaneous white adipose tissue (scWAT), and *Cpt1* was elevated in the perigonadal (pgWAT) tissue (Figure 5). In the female *Sigmar1−/−* (vs. *Sigmar1+/+*) mice, *Fatp4* was elevated in the scWAT and *Lpl* and *Mcad* were increased in the pgWAT. These changes were consistent with the reduced fat mass in the male and female *Sigmar1−/−* mice. By contrast, the female *Tmem97−/−* mice that had no change in fat mass showed a reduced expression of *Lpl* and *Mcad* in the scWAT, and a reduced expression of *Cpt1, Lcad,* and *Mcad* in the pgWAT (Figure 6). There was no appreciable change in gene expression in the male *Tmem97−/−* mice, except that the *Mcad* was decreased in the scWAT and the *Fatp4* was increased in the pgWAT. These changes could contribute to reductions in fat mass, but the mechanism is unclear.

## 3. Discussion

Sigma receptors have been studied pharmacologically for decades [3], yet little is known regarding their gene-specific role in obesity. Investigation of this topic may provide potential new therapeutic targets. Our data revealed that either S1R KO (in both sexes) or S2R KO (in male mice) resulted in protection against HFD-induced adiposity. However, S2R ablation, but not S1R depletion, increased fatty-acid oxidation, suggesting that the underlying mechanisms are different. None of these changes were observed in the female S2R KO mice, indicating a sexually dimorphic phenotype.

In our recent report, we found that S1R ablation impeded body-weight gain in mice fed an HFD [15], but how this affected body composition was not known. Herein, our data show that while the animals’ fat mass markedly decreased, the lean mass increased in male and female S1R KO mice relative to the WT control groups and independently of food intake. Since the fat mass and lean mass changed in opposite directions, we inferred that the lower body weights observed in the S1R KO compared with the WT mice were likely due to lower fat mass. Interestingly, the S1R KO mice did not show changes in metabolism or energy expenditure, suggesting that the lower fat mass was not related to fat oxidation. However, adipogenesis could be impaired in S1R KO mice, as suggested by our recent in vitro data [15], which may partially explain the reduced fat mass. Therefore, the role of S1R in adipogenesis and the underlying mechanism deserve more detailed future studies.

Growing evidence indicates that S1R and S2R, though overlapping in ligand binding, differ substantially in their biological functions [3]. For instance, a recent study using knockout mice suggested that S1R and S2R played opposite roles in neuropathic pain perception [23]. Moreover, in pharmacological investigations, S1R and S2R appeared to have opposing roles in cell survival/death, although this conclusion needs to be genetically verified given that S2R ligand effects are not necessarily mediated by TMEM97 [24]. Herein, while we found that the deletion of either S1R or S2R protected against diet-induced adiposity, the underlying mechanisms appeared to be different. Unlike S1R KO mice, male S2R KO mice exhibited higher metabolic rates, as indicated by their increased O_2_ consumption and CO_2_ production. There are two major types of fuel for energy production: carbohydrates and fats. Interestingly, our further analysis indicated that the RER was reduced in the male S2R KO mice compared to the wild-type mice, indicating increased fatty-acid oxidation, and which was consistent with the reduction in fat mass. Another important factor contributing to fat mass is adipogenesis. Of note, while this manuscript was in preparation, Tenta et al. reported [25] that S2R/TMEM97 overexpression impaired adipogenesis in vitro, which implicated that S2R KO may enhance adipogenesis, which would not explain the reduction in fat mass. This further underscores the role of increased fatty-acid oxidation in S2R KO male mice. Nevertheless, more sophisticated studies are needed to elucidate the exact mechanism responsible for the protection against diet-induced adiposity afforded by S2R/TMEM97 ablation in male mice, with a focus on the pathways of fatty-acid oxidation.

It is also interesting to note that, unlike the male S2R KO mice, the female S2R KO mice showed no changes in metabolic measurements. This S2R-associated sexual dimorphism has not been previously reported, and may hold promise as a new research direction. In fact, few investigations have been conducted on the role of sigma receptors in gender-dependent physiological or pathobiological regulations [26]. On the other hand, sex hormones, such as progesterone, have long been suggested to be endogenous sigma-receptor ligands, although their binding affinities are generally low [27]. Other steroids and derivatives have also been reported to bind sigma receptors. For example, N,N dimethyl sphingosine bound to purified S1R with an affinity of ~100 nM [28]. Cholesterol was also found to bind to S1R [29,30], although it is not clear whether cholesterol occupies the S1R ligand-binding pocket recently elucidated in S1R crystal structures [9,31]. Interestingly, chemoproteomics and molecular docking revealed that the biologically active molecule 20(S)-hydroxycholesterol bound in a central binding pocket of TMEM97 [32], in accordance with the newly solved TMEM97 atomic structure [8] and previous reports on TMEM97 participating in cholesterol transport [20,21]. However, it remains unclear whether the observed sexual dimorphism relates to S2R’s cholesterol- and steroid-binding properties. Further research in this regard is warranted, especially in view of the fact that sexual dimorphism is increasingly appreciated for its broad influence in biology and pathology. Taken together, our results suggest a potentially important role for S1R and S2R in adipose-tissue biology, lipid metabolism, and obesity.

## 4. Materials and Methods

### 4.1. Animals

All animal procedures conformed with the NIH Guide for the Care and Use of Laboratory Animals and were in compliance with the Institutional Animal Care and Use Committee with approved protocols. Mice were maintained on a normal diet (Envigo Teklad 7912; 17% kcal fat and a total of 3.1 kcal/g) with standard light/dark cycles (12 h/12 h). Animals were euthanized in a chamber gradually filled with CO_2_.

### 4.2. Sigmar1−/− and Sigmar1+/+ Mice

The establishment of the *Sigmar1−/−* mouse colony was previously reported [10]. Briefly, a heterozygous Oprs1 (Sigmar1) mutant line (OprsGt (IRESBetageo) 33Lex) on a C57BL/6 J × 129 s/SvEv mixed background was purchased from the Mutant Mouse Regional Resource Center (MMRRC, UC, Davis, CA, USA). A relatively uniform background was reached through back-crossing to C57BL/6J mice (JAX#000664), which was repeated occasionally to refresh the colony and prevent genetic shift. While selecting *Sigmar1−/−* pups, littermate *Sigmar1+/+* mice were identified for use as wild-type controls. Genotyping was performed as we reported in [15].

### 4.3. Tmem97−/− and Tmem97+/+ Mice

As we recently reported [33], sperms of the C57BL/6N-Tmem97tm1.1 (KOMP)Vlcg strain were purchased from the KOMP Repository at UC Davis (stock#10753A-D5). The strain was revived against the background of C57BL/6J and backcrossed with C57BL/6J mice to attain a relatively uniform background. Homozygous *Tmem97−/−* and *Tmem97+/+* littermates from heterozygous breeders were used in experiments. Mice were genotyped following the protocol in our previous report [33].

### 4.4. High-Fat-Diet Feeding

We followed the conditions described in our previous report [15]. Briefly, at 8–10 weeks of age, mice were switched to HFD (Research Diets Inc. D12492; 60% kcal fat, 20% kcal carbohydrate, 20% kcal protein, 5.21 kcal/g). The chow was changed twice a week, and fragments or powder were avoided for accurate measurement of food intake. Live animal measurements (such as metabolism) were performed 6 weeks after HFD, and the animals were euthanized after feeding with HFD for 12 weeks or 13 weeks (specified in figure legends). Body weights were measured weekly throughout the time period of HFD feeding. In this study, we used eight groups of mice fed on HFD, including 7 male *Sigmar1−/−* mice, 8 male-littermate *Sigmar1+/+* mice, 8 female *Sigmar1−/−* mice, 8 female-littermate *Sigmar1+/+* mice, 10 male *Tmem97−/−* mice, 11 male-littermate *Tmem97+/+* mice, 10 female *Tmem97−/−* mice, and 10 female-littermate *Tmem97+/+* mice. The total number of mice was 72.

### 4.5. Assessments of Live Animal Body Composition

We used an EchoMRI analyzer (EchoMRI LLC) [34]. Since the NMR spectrum of canola oil is comparable to that of the fat in mice, we used the oil to calibrate the device. Each mouse was placed in a plastic tube and constrained with a stopper. The tube was then placed into the EchoMRI, and the total fat mass, total lean mass, and body weight were calculated. The fat and lean-mass percentages were calculated by dividing the total fat/lean mass by the total body mass.

### 4.6. Measurements of Whole-Body Metabolism

Measurements were performed after 6 weeks of HFD. Each mouse was placed in an individual metabolic cage and acclimatized for 24 h, after which measurements were carried out for 24 h in the fed state and 24 h in the fasted state. Serial measurements were made and the area under the curve (AUC) or average (for RER) of each 12-h time period was calculated for each individual animal. The Comprehensive Lab Animal Monitoring System (Oxymax Opto-M3; Columbus Instruments) was used to measure volume of O_2_ consumption (VO_2_), volume of CO_2_ production (VCO_2_), and energy expenditure [34]. The respiratory exchange ratio (RER) was calculated as VCO_2_/VO_2_. Energy expenditure was calculated using indirect calorimetry according to the equation (3.941 × VO_2_ + 1.106 × VCO_2_)/1000.

### 4.7. Biochemical Methods

Fat tissues were dissected from the mice that were euthanized at the end of HFD feeding. Tissue processing and qPCR were performed as previously described [35]. Sigma-Aldrich custom primers were used for genes of interest. Sequences are shown in Supplementary Table S5. RNA was extracted using TRIzol (Ambion, Austin, TX, USA) and reverse-transcribed using the High-Capacity cDNA Reverse Transcription Kit (Applied Biosystems, Waltham, MA, USA). The qPCR was performed using SsoAdvanced SYBR Green Supermix (Bio-Rad, Hercules, CA, USA) and data were analyzed using the delta-delta CT method. All qPCR gene expression was normalized to the housekeeping gene GAPDH.

### 4.8. Glucose-Tolerance and Insulin-Tolerance Tests

Glucose-tolerance tests (GTT) were performed 6 weeks after the onset of HFD feeding. Mice were fasted for 11 h (22:00–9:00) with free access to drinking water. A baseline blood sample was collected from the tails of fully conscious mice, followed by i.p. injection of glucose (2.0 g/kg body weight), and blood was taken from the tails at 15, 30, 60, and 120 min after injection. Insulin tolerance tests (ITTs) were performed 6 weeks after the onset of HFD feeding. Mice were fasted for 2 h (10:00–12:00), and baseline blood samples were collected from the tails of fully conscious mice. Insulin (1 U/kg body weight) (Humulin; Eli Lilly) was administered by i.p. injection, and blood samples were taken from the tails at 10, 15, 30, 45, and 60 min after injection. Glucose concentrations were determined using a OneTouch Ultra-portable glucometer (LifeScan).

### 4.9. Statistical Analysis

Statistical analysis was performed using Prism GraphPad (version 8, GraphPad Software, San Diego, CA, USA), by one-way analysis of variance (ANOVA) with Bonferroni’s multiple comparison tests. Unpaired two-tailed Student’s t tests were performed for two-group comparison, as specified in supplemental figure legends. Values of *p* < 0.05 were considered significant.

## 5. Conclusions

This study identifies new biological functions for sigma receptors. The ablation of S1R in both sexes or S2R deletion in male mice ameliorates HFD-induced obesity. While no changes in metabolism were observed in S1R KO mice, S2R ablation enhanced metabolism, fat oxidation, and insulin tolerance only in male mice. These results suggest differential S1R- and S2R-mediated mechanisms and sexual dimorphism, which may be targeted for precision intervention to tackle obesity. The importance of identifying targets to treat obesity is underlined by the fact that this disease predisposes patients to comorbidities, including type 2 diabetes and cardiovascular disease, that significantly increase mortality, morbidity, and financial healthcare burdens [1]. In this regard, the enhanced insulin tolerance in S2R KO mice (also observed by Tenta et al.) [25] is encouraging and suggests S2R as a potential therapeutic target to combat insulin resistance. On the other hand, our study also proposes possible caveats for the ongoing clinical translation targeting sigma receptors. For example, while S1R agonists have shown benefits in preclinical and clinical trials treating an array of diseases [3,18,19], a possible effect of these drugs on body composition and glucose tolerance may be considered. Therefore, to aid the design of effective and safe treatments targeting sigma receptors, more research is required to attain a better understanding of the functions of S1R and S2R and the mechanisms underlying them.

## Supplementary Materials

The following supporting information can be downloaded at: https://www.mdpi.com/article/10.3390/ijms231810846/s1.
