# Supplementary material for: Differential Responses to Sigma-1 or Sigma-2 Receptor Ablation in Adiposity, Fat Oxidation, and Sexual Dimorphism
Source: Int J Mol Sci. 2022 Sep 16;23(18):10846. doi: 10.3390/ijms231810846 (PMC9506228; doi:10.3390/ijms231810846)
Supplement: Supplementary file 1 [file ijms-23-10846-s001.zip › ijms-1901885-supplementary.pdf]

International Journal of
Molecular Sciences

MDPI

Supplemental Figures

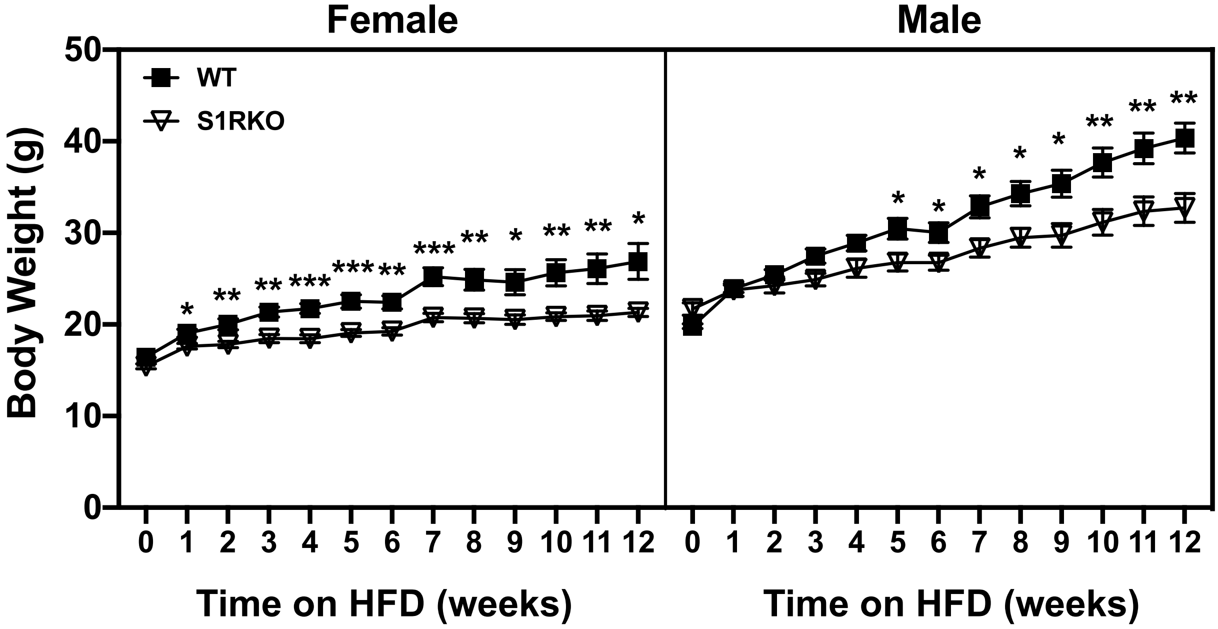

Figure S1. Time course of body weight throughout the period of HFD feeding of S1R WT and KO mice
Body weight was measured weekly until the end of 12 weeks of HFD. Statistical analysis: Unpaired two-tailed Student's *t* test for each time point (*P < 0.05, **P <0.01, ***P<0.001), S1R KO *vs* WT, n = 7 or 8 mice. Data are presented as means ± SE. False discovery rates (FDRs) are presented in Table S1 and S2.

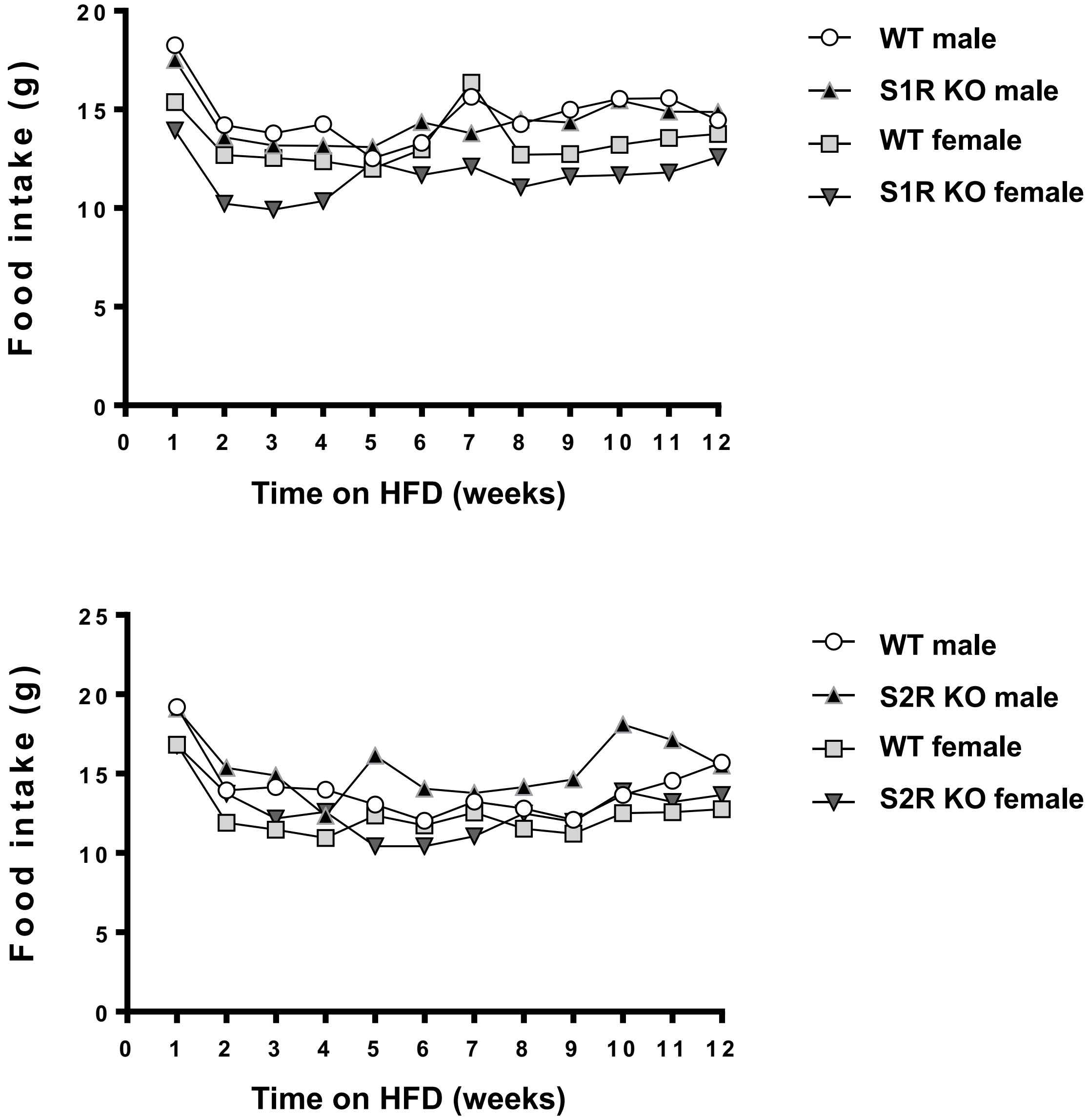

Figure S2. Food intake during the period of HFD feeding

Each data value represents averaged food intake per mouse, i.e. one week of food intake divided by animal number (4 or 5 mice in a cage). The time course indicates 12 weeks of HFD.

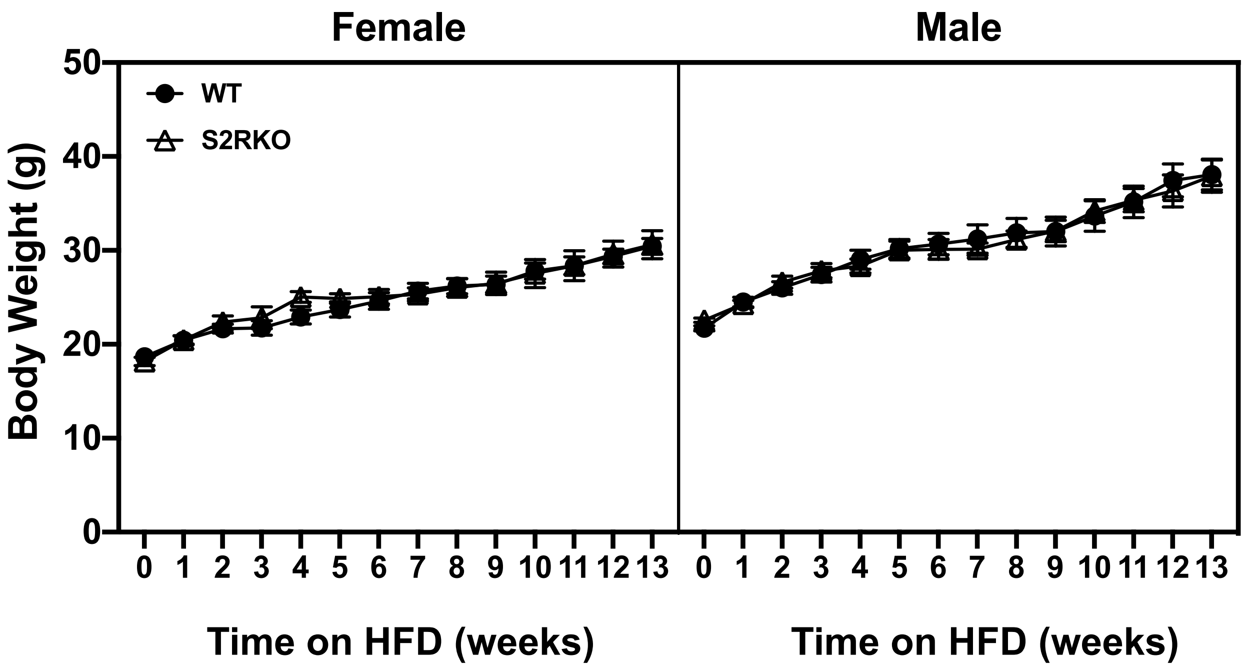

Figure S3. Time course of body weight throughout the period of HFD feeding of S2R WT and KO mice Body weight was measured weekly until the end of 13 weeks of HFD. Statistical analysis: Unpaired two-tailed Student's *t* test for each time point, S2R KO *vs* WT, n = 10 or 11 mice. No difference was detected (P>0.05). Data are presented as means ± SE. FDRs are presented in Table S3 and S4.

Supplementary Tables

Table S1. FDR caculated for female WT and S1R KO mice (see Figure S1 female).

WT-F vs S1RKO-F	sorted p value	q value	BH critical value (ranki/n)*0.05	Dis-cover?
week0	0.076095097	0.076095097	0.05	NO
week1	0.018417511	0.019952303	0.046153846	YES
week2	0.006728338	0.010933549	0.030769231	YES
week3	0.001079284	0.003507672	0.015384615	YES
week4	0.000230513	0.002996668	0.003846154	YES
week5	0.00084544	0.005495359	0.007692308	YES
week6	0.001724685	0.00448418	0.019230769	YES
week7	0.000961821	0.00416789	0.011538462	YES
week8	0.004401672	0.009536956	0.023076923	YES
week9	0.014388824	0.018705472	0.038461538	YES
week10	0.006100423	0.011329357	0.026923077	YES
week11	0.009066376	0.013095876	0.034615385	YES
week12	0.015167965	0.017925777	0.042307692	YES

Table S2. FDR caculated for male WT and S1R KO mice (see Figure S1 male).

WT-M vs S1RKO-M	p value	q value	BH critical value (ranki/n)*0.05	Dis-cover ?
week0	0.01734927	0.03222008	0.02692308	Yes
week1	0.88001584	0.88001584	0.05	NO
week2	0.24078743	0.26085305	0.04615385	NO
week3	0.02846692	0.04111889	0.03461538	Yes
week4	0.05024536	0.05938088	0.04230769	NO
week5	0.02795588	0.04542831	0.03076923	Yes
week6	0.03399045	0.04418759	0.03846154	Yes
week7	0.01283047	0.04169903	0.01538462	Yes
week8	0.01512892	0.03277932	0.02307692	Yes
week9	0.01370901	0.03564342	0.01923077	Yes
week10	0.00966085	0.06279552	0.00769231	Yes
week11	0.01091571	0.04730142	0.01153846	Yes
week12	0.00518944	0.06746275	0.00384615	Yes

Table S3. FDR caculated for female WT and S2R KO mice (see Figure S3 female).

WT-F vs S2RKO-F	sorted p value	q value	BH critical value (ranki/n)*0.05	Dis-cover?
week 0	0.37544707	1.75208634	0.010714286	NO
week 1	0.94316191	1.10035557	0.042857143	NO
week 2	0.40833802	1.42918306	0.014285714	NO
week 3	0.45008804	1.26024652	0.017857143	NO
week 4	0.03619417	0.50671837	0.003571429	NO
week 5	0.23741862	1.66193031	0.007142857	NO
week 6	0.70408195	1.64285788	0.021428571	NO
week 7	0.75051461	1.50102921	0.025	NO
week 8	0.90469602	1.26657443	0.035714286	NO
week 9	0.9480797	1.02100891	0.046428571	NO
week 10	0.8825426	1.54444955	0.028571429	NO
week 11	0.99745229	0.99745229	0.05	NO
week 12	0.88502655	1.37670797	0.032142857	NO
week 13	0.9239	1.17587273	0.039285714	NO

Table S4. FDR caculated for male WT and S2R KO mice (see Figure S3 male).

WT-M vs S2RKO-M	sorted p value	q value	BH critical value (ranki/n)*0.05	Dis-cover?
week 0	0.02620175	0.36682454	0.00357143	NO
week 1	0.75239154	1.17038683	0.03214286	NO
week 2	0.5128006	3.58960417	0.00714286	NO
week 3	0.70196474	1.40392947	0.025	NO
week 4	0.60055042	2.10192647	0.01428571	NO
week 5	0.91527219	1.16489187	0.03928571	NO
week 6	0.71803228	1.2565565	0.02857143	NO
week 7	0.537697	2.50925268	0.01071429	NO
week 8	0.70097354	1.63560494	0.02142857	NO
week 9	0.98063818	0.98063818	0.05	NO
week 10	0.80290611	1.12406856	0.03571429	NO
week 11	0.93072309	1.08584361	0.04285714	NO
week 12	0.65074127	1.82207555	0.01785714	NO
week 13	0.94837023	1.02132178	0.04642857	NO

Table S5. Primer sequences for qPCR.

	Mouse (5' to 3')	
**Gene symbol**	**Forward**	**Reverse**
Lpl	GATGCCCTACAAAGTGTTCCA	AAATCTCGAAGGCCTGGTTG
Fatp4	GACTTCTCCAGCCGTTTCCACA	CAAAGGACAGGATGCGGCTATTG
Fabp5	GACGACTGTGTTCTCTTGTAACC	TGTTATCGTGCTCTCCTTCCCG
Cd36	TGGAGCTGTTATTGGTGCAG	TGGGTTTTGCACATCAAAGA
Cpt1	AAAGATCAATCGGACCCTAGACA	CAGCGAGTAGCGCATAGTCA
Lcad	TTTCCGGGAGAGTGTAAGGA	ACTTCTCCAGCTTTCTCCCA
Mcad	GATGCATCACCCTCGTGTAAC	AAGCCCTTTTCCCCTGAA
Pdk4	GTCGAGCATCAAGAAAACCGTCC	GCGGTCAGTAATCCTCAGAGGA
Hsl	GCTCATCTCCTATGACCTACGG	TCCGTGGATGTGAACAACCAGG
